# Machine Learning in the Detection of Oral Lesions With Clinical Intraoral Images

**DOI:** 10.7759/cureus.44018

**Published:** 2023-08-24

**Authors:** Dinesh Y, Karthikeyan Ramalingam, Pratibha Ramani, Ramya Mohan Deepak

**Affiliations:** 1 Oral Pathology and Microbiology, Saveetha Dental College and Hospitals, Saveetha Institute of Medical and Technical Sciences, Saveetha University, Chennai, IND; 2 Engineering, Saveetha School of Engineering, Saveetha Institute of Medical and Technical Sciences, Saveetha University, Chennai, IND

**Keywords:** ai & robotics in healthcare, ai and robotics in healthcare, clinical images, artificial intelligence, oral potentially malignant disorders, oral cancer, machine learning

## Abstract

Introduction: Artificial intelligence in oncology has gained a lot of interest in recent years. Early detection of Oral squamous cell carcinoma (OSCC) is crucial for early management to attain a better prognosis and overall survival. Machine learning (ML) has also been used in oral cancer studies to explore the discrimination between clinically normal and oral cancer.

Materials and methods: A dataset comprising 360 clinical intra-oral images of OSCC, Oral Potentially Malignant Disorders (OPMDs) and clinically healthy oral mucosa were used. Clinicians trained the machine learning model with the clinical images (n=300). Roboflow software (Roboflow Inc, USA) was used to classify and annotate images along with Multi-class annotation and object detection models trained by two expert oral pathologists. The test dataset (n=60) of new clinical images was again evaluated by two clinicians and Roboflow. The results were tabulated and Kappa statistics was performed using SPSS v23.0 (IBM Corp., Armonk, NY).

Results: Training dataset clinical images (n=300) were used to train the clinicians and Roboflow algorithm. The test dataset (n=60) of new clinical images was again evaluated by the clinicians and Roboflow. The observed outcomes revealed that the Mean Average Precision (mAP) was 25.4%, precision 29.8% and Recall 32.9%. Based on the kappa statistical analysis the 0.7 value shows a moderate agreement between the clinicians and the machine learning model. The test dataset showed the specificity and sensitivity of the Roboflow machine learning model to be 75% and 88.9% respectively.

Conclusion: In conclusion, machine learning showed promising results in the early detection of suspected lesions using clinical intraoral images and aids general dentists and patients in the detection of suspected lesions such as OPMDs and OSCC that require biopsy and immediate treatment.

## Introduction

Oral cancer contributes to 90% of head and neck malignancies. Oral squamous cell carcinoma (OSCC) is the sixth most common malignancy in the world; early detection and management are essential for a good prognosis and survival rate. Despite the advancements in diagnosis and management of oral cancer, most of the OSCC cases are diagnosed in advanced disease stages, stage III and stage IV. The estimated five-year survival rate for OSCC demonstrates a significant decrease in advanced stages [1,2]. 

Oral Potentially Malignant Disorders (OPMDs) are oral mucosal abnormalities with an increased risk of developing oral cancer. In India, several risk factors such as smokeless tobacco, smoking, alcohol consumption, and lifestyle are commonly found to be associated with OPMDs such as oral submucous fibrosis, oral leukoplakia, oral erythroplakia, etc. Early detection of OPMDs is crucial because of their high malignant transformation rate [3,4]. 

Diagnosis of OSCC and OPMDs totally relies on invasive biopsy procedures and histopathology; advances in technology have several innovations in prediction such as vital staining, light-based imaging-chemifluorescence, liquid biopsy for molecular biomarkers, etc. Artificial intelligence (AI) is commonly employed in diverse areas of medicine. Digitalization and machine learning have gained acceptance in the field of medicine. Traditional machine learning employs algorithms and computer processes to calculate data and identify input data patterns in order to provide a quantitative diagnostic result [5,6]. 

Machine learning in artificial intelligence shows significant development and acceptance among researchers. The need for accuracy in the diagnosis of OSCC and OPMDs using clinical data is crucial for increasing survival rates. Advances in computer vision and deep learning provide potent methods for developing supplementary technologies that can perform an automated oral cavity screening. Vision-based adjunctive technologies offer opportunities for oral cancer and OPMD screening processes [7]. Machine learning provides opportunities for oral cancer and OPMD screening [8].

Machine learning (ML) has also been used in oral cancer studies to explore the discrimination between clinically normal and oral cancer. Optimistic performance, conventional machine learning-related classification lacks the capacity to quantify decision uncertainty for physicians. In this study, an ML model was developed for classifying images of normal healthy mucosa and suspected cases of oral cancer or oral potentially malignant disorders. This structure measures the uncertainty of the methods' outputs and suggests cases with higher uncertainty values for further analysis. This machine learning predicts the trained annotated sites and is designed to complement existing medical methods [9].

This study aimed to explore the potential application of machine learning using clinical images to investigate the sensitivity and specificity of an automated system for detecting oral lesions. The objectives were the collection of clinical images of Oral cancer, OPMDs, and clinically normal lesions, the annotation of the clinical images using labels, validation and testing of machine learning models in clinical diagnosis.

## Materials and methods

The study was conducted in the Department of Oral Pathology, Saveetha Dental College and Hospitals, Chennai. We retrospectively collected photographic intraoral images taken using a mobile camera or iPad with resolution ranging between 4 MP to 16 MP. No transformation or editing of the images was done. Previously diagnosed cases under clinical examination by experts and OPMDs and OSCC cases were retrieved from the Dental Information Archived Software (DIAS) from January 2021 to March 2023.

The data utilized were clinical intra-oral images (n=360) of OSCC, OPMDs, and non-pathological oral images from different areas of the oral cavity such as the buccal mucosa, tongue, upper/lower alveolar ridge, floor of the mouth, retromolar trigone, and lip. The dataset used for lesion and normal mucosa detection consisted of 360 patients with one digital photograph of each patient. Specifically, (n=300) of the images were used for training and (n=60) for testing purposes. The images in this study varied in resolution, with the largest image measuring 4501 x 2986 pixels and the smallest image measuring 1120 x 821 pixels. The dataset included a diverse range of lesions resulting from various oral diseases and anatomical regions. Clinically normal oral images represent the oral mucosa without any clinically identifiable lesions.

Clinical picture evaluation by subject experts vs. machine learning model

All the images utilized in the study were histopathologically confirmed for clinically normal mucosa, OPMDs, and OSCC. The retrieved clinical images were again evaluated by two subject experts and Roboflow software (Roboflow Inc, USA). The training session included 300 images for both groups. The testing session included 60 images to verify the concordance between both groups.

**Figure 1:**
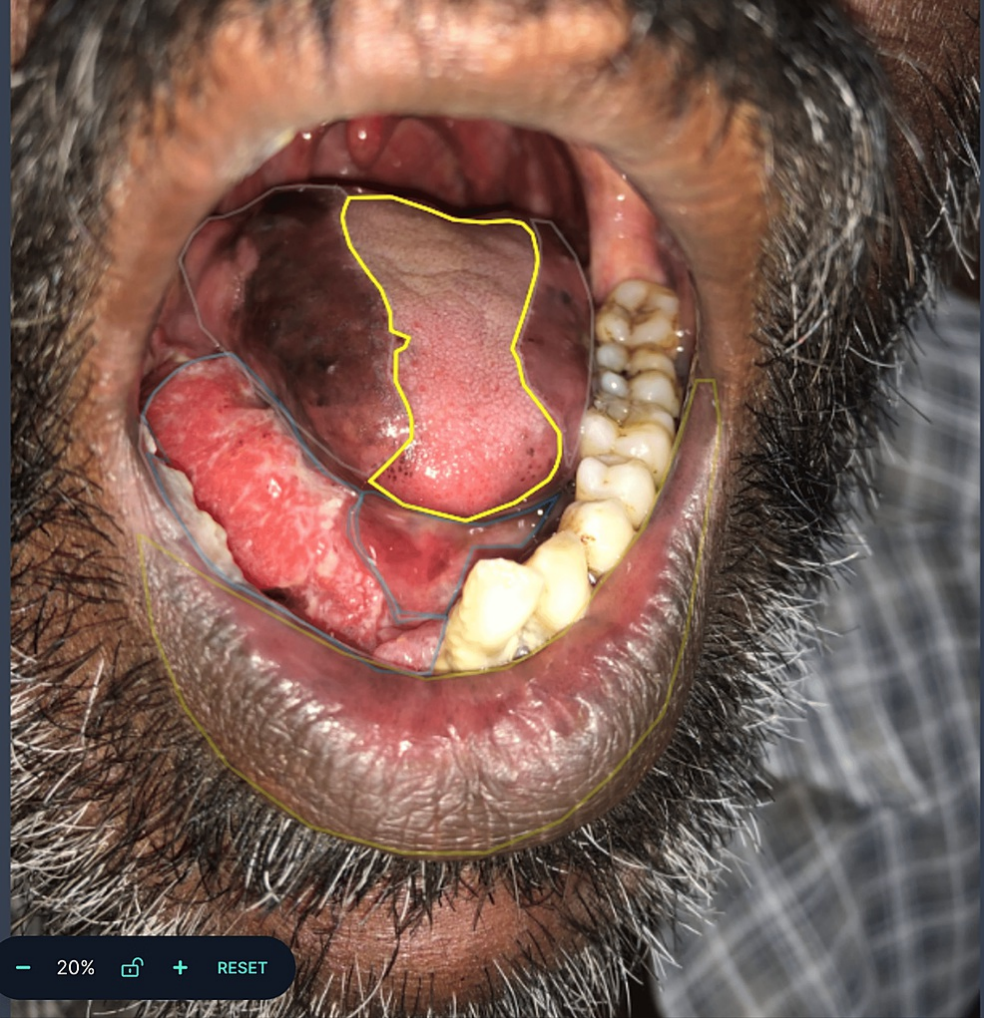
Image representing annotation of the clinical intraoral image using Roboflow software Roboflow software (Roboflow Inc, USA)

**Figure 2:**
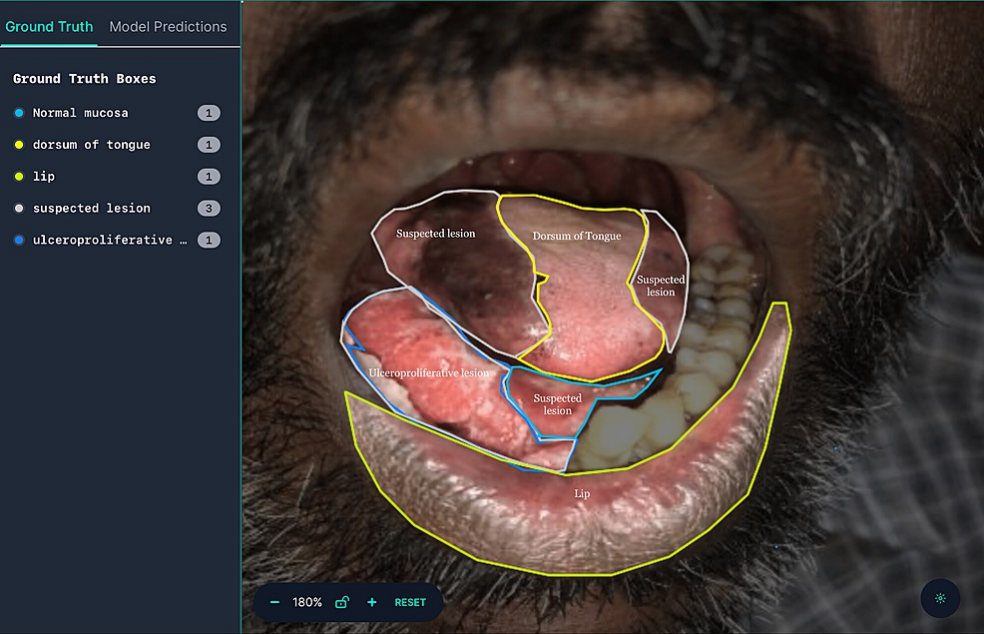
Image representing object detection and labelling of the clinical intraoral image using Roboflow software Roboflow software (Roboflow Inc, USA)

The study focused on analyzing images of OSCC and OPMDs presented as white and red lesions in the form of patches, ulcers, striae, plaques, etc. by clinicians and Machine Learning. The test set (n=60) was blinded and two expert oral and maxillofacial pathologists analyzed the images to categorize them as normal mucosa and suspected lesions. Roboflow software was used to classify and annotate images with multiclass annotation and object detection models. As per instructions in Roboflow tutorials, the model was trained by two expert oral pathologists utilizing 300 images. The lesion was annotated by outlining them with polygons and assigning corresponding class values as region attributes (Figures 1, 2). Precision and recall values were calculated. 

Precision = True positive/ True positive+False positive

Recall = True positive/True positive/False negative

Image classification based on color: grey lesion, white lesion, red lesion, red and white lesion and brown hyperpigmentation; based on clinical presentation: patch, ulcer and ulceroproliferative lesion; anatomical structures: lip, skin, dorsum of the tongue. The structures were finally annotated for normal mucosa and suspected mucosa. Statistical analysis was done using Kappa statistics with SPSS software v23.0 (IBM Corp., Armonk, USA).

## Results

**Figure 3:**
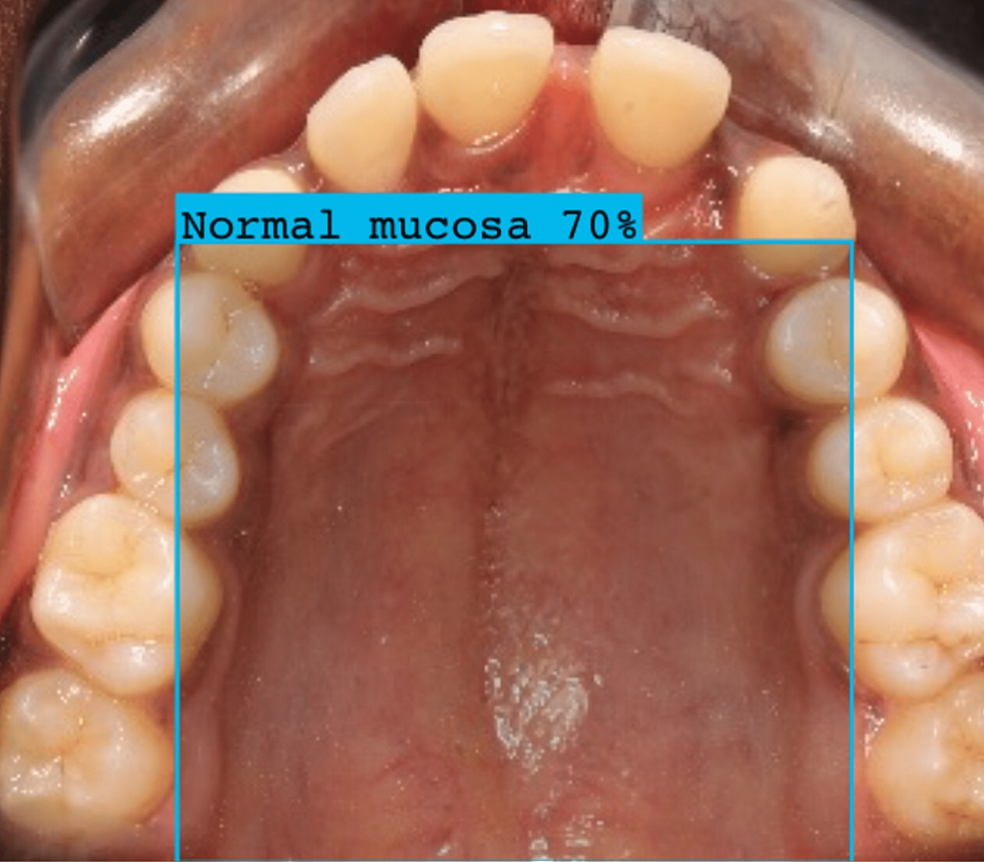
Image represents normal mucosa detection using Roboflow software Roboflow software (Roboflow Inc, USA)

**Figure 4:**
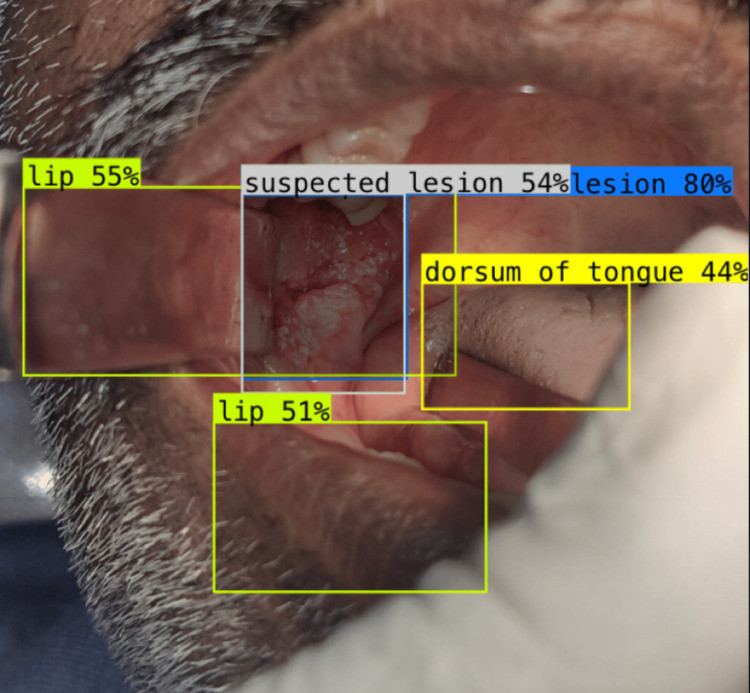
Image represents suspected lesion detection using Roboflow software Roboflow software (Roboflow Inc, USA)

**Figure 5:**
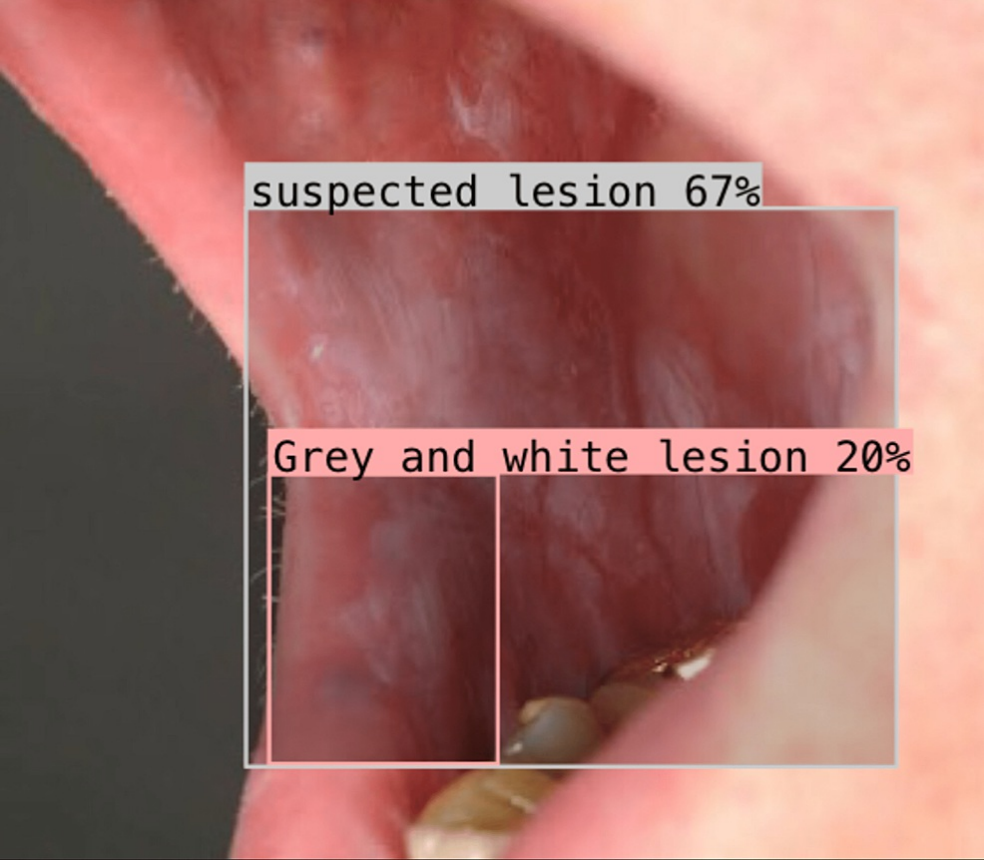
Image represents OPMD suspected lesion detection using Roboflow software Roboflow software (Roboflow Inc, USA)

We constructed a dataset of 360 images, which was clinically and histopathologically examined for normal mucosa and suspected lesions. The dataset was blinded and a test set (n=60) was analysed by two experts using the images and compared the results with that of Roboflow software. The object detection using Roboflow software for normal mucosa, OPMD, and OSCC is presented in Figures 3-5.

Testing for precision

**Figure 6:**
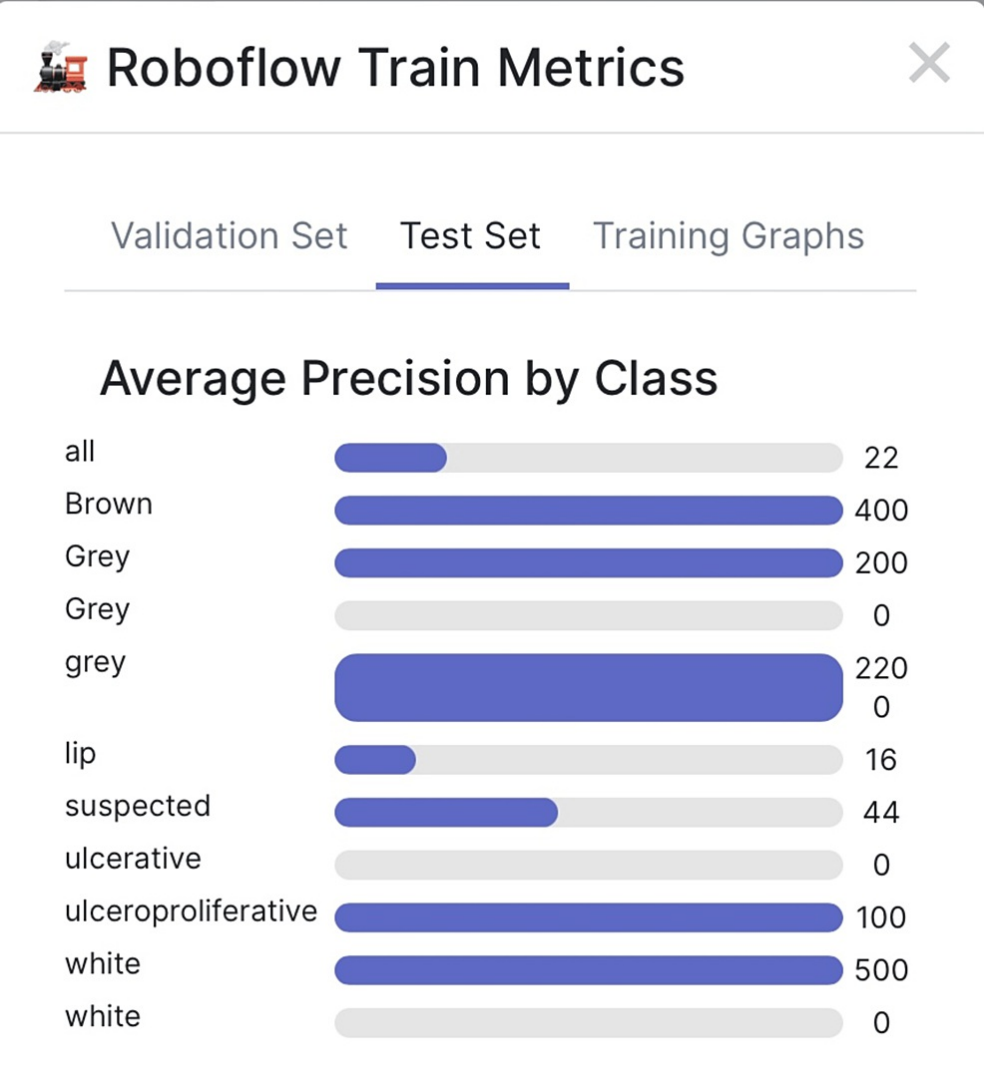
Image represents average precision in the validation set

**Figure 7:**
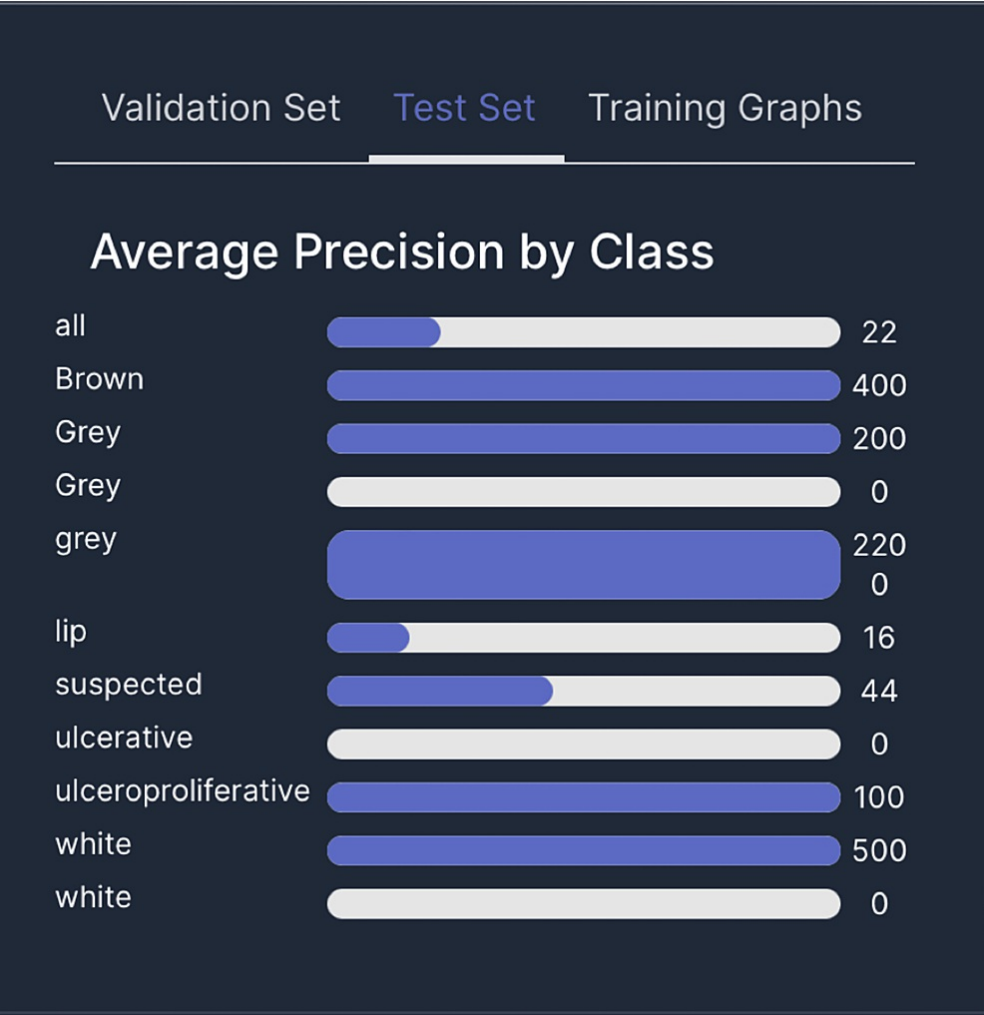
Image represents average precision in the test set

The Mean Average Precision (mAP) was 25.4%, precision 29.8% and Recall 32.9%. Average precision among validated sets by class showed high precision found in grey lesions, gingival brown pigmentation, white lesions, ulceroproliferative, and suspected lesions (Figure 6). The test set classes showed precision in the identification of white lesions, grey lesions, ulcerative, ulceroproliferative lesions, and normal mucosa-dorsum of the tongue, brown pigmentation, and skin (Figure 7). Average precision among the test set showed high precision in white, grey patch lesions, brown hyperpigmentation, ulceroproliferative lesions and suspected lesions.

Machine learning vs. subject experts

The specificity and sensitivity of the Roboflow Machine Learning model were 75% and 88.9% respectively (Table 1). The true positive results of clinically healthy oral mucosa by the subject experts were 100% and the Roboflow model was 80%. The true positive results of suspected lesions (OPMDs & OSCC) by the subject experts was 90% and the Roboflow model was 85%. Based on the Kappa statistical analysis, the 0.7 value showing a moderate agreement between the blinded subject experts and the Roboflow model was almost perfect (Table 2).

**Table 1:** The percentage of agreement between the subject experts and the Roboflow model The comparison and the standard error refer to cases with false results. OPMD: Oral potentially malignant disorder; OSCC: Oral squamous cell carcinoma

Groups	Subject Experts	Standard error	Roboflow model	Standard error
Clinically healthy oral mucosa (n=20)	100%	0	80%	20%
Suspected lesions (OPMDs & OSCC) (n=40)	90%	10%	85%	15%

**Table 2:** Kappa statistics result The table represents kappa statistics of value 0.7 - a moderate agreement between the Subject Experts and the Roboflow model.

Symmetric Measures	N of Valid Cases	Kappa Value	Asymptotic Standardised Errors	Approximate Tb	Approximate Significance
Measure of Agreement	60	0.71	0.143	3.557	0

## Discussion

Several novel methods are evolving in the field of diagnosis of cancers such as machine learning, exosomes and paper-based biosensors [10]. Oncology is being revolutionized by cutting-edge machine learning algorithms and provides rapid and cutting-edge methods for the diagnosis of potential lesions with an accuracy comparable to that of medical specialists. Despite the easy accessibility of the buccal cavity during regular examinations, numerous malignancies remain undetected until they reach advanced stages. Consequently, employing AI holds promise in addressing the high mortality rates linked to oral cancer. The increasing availability of medical digital data makes the progress of AI-driven image analysis at a rapid rate. This advancement shows the potential to enhance the quality of life of patients [11]. 

In their systematic review, Mahmood et al. studied AI-based approaches for diagnosing head and neck cancer, encompassing various imaging modalities; histopathological (n=9) and radiological (n=8) were the common modalities studied [12]. Additionally, two investigations incorporated clinicopathologic/genomic information. Among the studies reviewed, 69 % of studies were ML methods, 25% of studies were deep learning (DL) methods, and 6% of studies had a combination of both. This demonstrates a growing number of studies on AI/ML in detecting head and neck cancer, utilising diverse imaging modalities [12]. 

In our investigation, the multiclass model was used to categorize the images since the images should be understandable even to a non-medical individual; the Roboflow machine learning model's specificity and sensitivity were 75% and 88.9%, respectively. A systematic review by Al Rawi et al. on supervised machine learning in oral cancer using clinical images revealed an accuracy range of 43.5% to 100%, a sensitivity of 94% to 100%, a specificity of 16% to 100%, and an area under the curve (AUC) of 93% was reported [8]. Similarly, a systematic review based on histopathological images demonstrated an accuracy between 89.47% and 100%, sensitivity between 97.76% and 99.26%, and specificity between 92% and 99.42% [13].

The study created an artificial intelligence algorithm using Roboflow, which demonstrated satisfactory performance with a mean average precision (mAP) of 25.4%. While certain previous medical imaging studies have achieved better results in specific instances compared to our AI model's performance validation, this diagnostic tool based on artificial intelligence holds potential clinical importance in clinical diagnosis. Object identification and annotation have been heterogeneous among previous studies; the predominantly clinical presentation of the lesion is labelled. This reduction in precision might be because the collected photographs were taken on a phone or iPad, a professional digital camera or an endoscope device can give better clarity of the lesions and further studies are planned on this topic. Recall value was low in our study compared to previous studies. It might be because of labelling of lesions as suspected lesions were based on clinical practice presentation rather than OPMD, OSCC, and normal.

A large, validated data set is one of the prerequisites for enhancing diagnostic performance despite recent innovations in deep learning technology. The Frankenstein dataset consists of information culled from multiple sources and pieced together. Likewise, because of the heterogeneity of the clinical presentation of OPMDs and OSCC several sources and a multicentric approach with collaboration with several other cancer institutes must be considered [14]. Object detection models such as convolutional neural network (CNN)-based models showed similar accuracy in recall between OPMD and OSCC [15].

The future scope of research on histopathological samples of OPMDs and OSCC is warranted to be implemented in whole slide imaging. Further, an interdisciplinary approach with oral physicians and oral surgeons with multi-centric images will produce more robust algorithms. We are currently pursuing a larger dataset with site-specific and disease-specific images to improve our current Roboflow algorithm. We are also pursuing the development of a mobile application that will help dental and non-medical professionals to identify suspected lesions and referral to higher centers for prompt management.

Limitations

Another limitation is the collection of images of varying resolutions of mobile cameras. Even physiologically altered conditions like Fordyce granules were identified as suspected lesions. The algorithm cannot make definite predictions for specific OPMDs and their potential for malignant transformation which is similar to subject experts unless specific parameters are assessed.

## Conclusions

We conclude that there is a potential for automatic identification of OPMDs and OSCC patients using machine learning approaches, with performance on par with or exceeding that of qualified human experts. To facilitate early detection and prompt referral, the devised algorithm with strong generalization potential might be employed as a convenient, non-invasive, and cost-effective tool. Machine learning showed a reliable detection tool in suspected lesions. Therefore this tool can assist general dentists in the early detection of suspected lesions.
